# Predictors of problematic smartphone use among university students

**DOI:** 10.1186/s41155-020-00147-8

**Published:** 2020-05-19

**Authors:** Paulo Guirro Laurence, Yuri Busin, Helena Scoz da Cunha Lima, Elizeu Coutinho Macedo

**Affiliations:** 1 Social and Cognitive Neuroscience Laboratory and Developmental Disorders Program, Center for Health and Biological Sciences, Mackenzie Presbyterian University, São Paulo, Brazil; 2 Social and Cognitive Neuroscience Laboratory of Mackenzie Presbyterian University, Rua Piaui, no. 181, 10th floor, São Paulo, 01241-001 Brazil

**Keywords:** Mobile phone, Cell phone, Addictive behavior, Addiction, Social network, Loneliness

## Abstract

Predictors of problematic smartphone use have been found mainly in studies on elementary and high school students. Few studies have focused on predictors related to social network and messaging apps or smartphone model. Thus, the objective of our study was to identify predictors of problematic smartphone use related to demographic characteristics, loneliness, social app use, and smartphone model among university students. This cross-sectional study involved 257 Brazilian university students who answered a smartphone addiction scale, a questionnaire about smartphone usage patterns, and the Brazilian version of the UCLA-R loneliness scale. Women, iPhone owners, and users of Instagram and Snapchat had significantly higher smartphone addiction scores. We found correlations between scores for the Brazilian version of smartphone addiction scale and the importance attributed to WhatsApp, Facebook, Instagram, and Snapchat, and the Brazilian version of the UCLA-R loneliness scale. Our hierarchical regression model predicted 32.2% of the scores of the Brazilian version of the smartphone addiction scale, with the greatest increase in predictive capability by the step that added smartphone social app importance, followed by the step that added loneliness. Adding the smartphone model produced the smallest increase in predictive capability. The theoretical and practical implications of these results are discussed.

## Introduction

Technological progress is one of the hallmarks of the new millennium. The use of smartphones has increased substantially over time, in part due to the many apps that these devices can support (Andrew, 2018). Although smartphones can be very useful and benefit the lives of many people, they can also bring problems such as “smartphone addiction” (Alosaimi, Alyahya, Alshahwan, Al Mahyijari, & Shaik, 2016; Boumosleh & Jaalouk, 2017). The term “addiction” is used when a person’s obsession with a certain activity is problematic to one’s daily life; smartphone addiction has patterns that are similar to substance dependency (Kwon, Kim, Cho, & Yang, 2013). For example, smartphone restriction can cause withdrawal symptoms (Eide, Aarestad, Andreassen, Bilder, & Pallesen, 2018).

Currently, the accuracy of the term “smartphone addiction” is being questioned. Panova and Carbonell (2018) explained that smartphone addiction is not really an addiction because crucial characteristics of an addiction are not achieved in the smartphone addiction construct. Examples of the lacking characteristics include the following: (1) the absence of severe physical consequences—one important characteristic of an addiction—as smartphone users have at most wrist and neck pain; (2) salience—the concept that the activity of addiction becomes the most relevant activity of the addicted—may not be true in smartphone addiction because smartphones mediate the social, professional, and personal lives of the user; (3) lack of longitudinal studies that confirm stability of the addiction as well as relapses, which are important aspects of addiction; and (4) smartphone addiction can be better explained by other conditions, such as an insecure attachment style, reassurance behavior, and other conditions, while a true addiction is not better explained by another condition. Panova and Carbonell (2018) also suggest that the smartphone may not be the addiction element, but rather the object related to an addiction; they compare the smartphone with the glass in alcohol addiction or the needle in heroin addiction. Further, Veissière and Stendel (2018) contend that “smartphone addiction” is not a real addiction, but simply a human desire to connect with other humans. As a result, Panova and Carbonell (2018) suggest abandoning the terminology of “smartphone addiction” and using “problematic smartphone use” instead, at least until there is more evidence to confirm the existence of a smartphone addiction.

Montag, Wegmann, Sariyska, Demetrovics, and Brand (2019) proposed that problematic smartphone use is essentially a type of Internet use disorder. In this sense, Internet use disorder should be divided into two areas: predominantly mobile and predominantly non-mobile. This new categorization may help problematic smartphone use to be recognized in the new International Classification of Diseases, under the umbrella of Internet use disorder, and accept the idea that the smartphone is just a way to use Internet, and not the problem itself.

A model was created to explain the interaction between several individual characteristics with Internet use disorder. The model is called “I-PACE” and is an acrogram for Interaction of Person-Affect-Cognition-Execution. This model accounts for biopsychological and social features of the person (e.g., genetics, personality, psychopathologies, motives to use), affect and cognition (e.g., attention, mood regulation, coping), and executive functions (e.g., inhibitory control, working memory (Brand, Young, Laier, Wölfling, & Potenza, 2016; Brand et al., 2019)).

Several studies have investigated the reasons for and the impact of excessive smartphone use, as well as potential risk factors for this behavior (e.g., Oviedo-Trespalacios, Nandavar, Newton, Demant, & Phillips, 2019; Lee, Kim, & Choi, 2017). In Australia, Oviedo-Trespalacios et al. (2019) found that there are more cases of problematic smartphone use now than the number of mobile phone users in 2005, particularly in 18–25-year-olds. In a study involving Korean adolescents, Lee and Lee (2017) reported that being female, focusing too much on the device, and having conflicts in real life due to excessive and ubiquitous smartphone use were risk factors for problematic smartphone use, while use of the device for learning was a protective factor. Others have also reported that women present more problematic smartphone use than men (Kwon, Kim, et al., 2013; Lapointe, Boudreau-Pinsonneault, & Vaghefi, 2013). Family income as a possible risk factor for problematic smartphone use has also been investigated by several researchers, but most research found no significant association (Aljomaa, Al Qudah, Albursan, Bakhiet, & Abduljabbar, 2016; Alhassan et al., 2018; Cha & Seo, 2018), with one exception (Aktürk, Budak, Gültekin, & Özdemir, 2018), although this study was not specifically designed to investigate this association. Moreover, Sanal and Ozer (2017) reported no correlation between university students’ major and problematic smartphone use.

The relationship between problematic smartphone use and psychological dysfunctions, including loneliness, has also been investigated. A recent study (Aktürk et al., 2018) reported an association between loneliness and problematic smartphone use among high school students, and other studies (Bian & Leung, 2014; Darcin, Noyan, Nurmedov, Yilmaz, & Dilbaz, 2015) have found this association in university students. However, other studies found no connection between loneliness and problematic smartphone use among university students (Darcin et al., 2016; Aktürk et al., 2018), requiring further research. Problematic smartphone use has been linked to anxiety, depression, low conscientiousness, and high neuroticism (Elhai et al., 2019; Peterka-Bonetta, Sindermann, Elhai, & Montag, 2019). Problematic smartphone use has also been connected with deficits in inhibitory control (Chen, Liang, Mai, Zhong, & Qu, 2016), attention, numerical processing, and the excitability of the right prefrontal cortex (Hadar et al., 2017). Additionally, Fransson, Chóliz, and Håkansson (2018) found that smartphone use and problem gambling are sometimes related, although further studies are needed.

One important element related to smartphone use is the fear of missing out (Elhai et al., 2019; Elhai, Yang, Fang, Bai, & Hall, 2020; Oberst, Wegmann, Stodt, Brand, & Chamarro, 2017; Wegmann, Oberst, Stodt, & Brand, 2017). Montag, Lachmann, Herrlich, and Zweig (2019) suggest that smartphone apps have a list of mechanisms that makes users use their apps even more. For social media, one of the most important mechanisms is using the fear of missing out. In this way, a user would be afraid of missing a friend’s reply to a message, thus checking the app more often. Another possibility would be observing their friends using an app and having fun, creating a social pressure for them to use it as well.

Differences in the characteristics of users of different models of smartphones (e.g., iPhone vs. Samsung; iOS vs. Android) have also been investigated, with conflicting results. While Shaw, Ellis, Kendrick, Ziegler, and Wiseman (2016) reported that iPhone users were more likely to be women, to be younger, and to view their smartphones as status symbols, Götz, Stieger, and Reips (2017) found few differences in personality between iOS and Android users. A survey involving 200 Stanford University students who used an iPhone reported that 10% of the participants demonstrated problematic smartphone use, 34% were likely to develop problematic use of the device, 69% stated that they were more likely to forget their wallet than their iPhone, and 41% said that it would be “a tragedy” if they lost their smartphone (Hope, 2010).

There has also been research on the social networks accessed by smartphone users because of concerns about their problematic use of networks (Kuss & Griffiths, 2017), especially for Instagram users (Huang & Su, 2018; Kicaburun & Griffiths, 2018). Instagram users state that they are motivated to access this network because they want to view posts and become involved in social interaction (Huang & Su, 2018). Kicaburun and Griffiths (2018) found a negative correlation between self-liking and Instagram problematic use and reported that users who spend more daily time on the Internet were the ones with the most problematic use. However, time spent on a smartphone should not be a criterion of problematic smartphone use, given that other motivations (e.g., work-related) can increase smartphone use (Billieux et al., 2015; King, Herd, & Delfabbro, 2018). Similarly, this caution should also be applied to using the number of messages as criterion (Panova & Carbonell, 2018). Several studies have examined the negative effects of the overuse of social networks and found that overuse is linked with depression, difficulty communicating face-to-face, need for immediate rewards, neglect of offline relationships, problems in professional contexts, and loneliness (for a review, see Kuss & Griffiths, 2017). Indeed, loneliness has been associated with social network addiction (De Cock et al., 2014). Similarly, Primack et al. (2017) found that individuals who were in the highest quartile of social media use were twice as likely to feel socially isolated.

Although accessing social networks has been singled out as the most used function in smartphones (Haug et al., 2015), few studies have investigated the relationship between problematic smartphone use and the importance of social networks. Most studies have only investigated whether individuals use social networks, and if so, the daily amount of time that they spend on social networks (Arnavut, Nutri, & Direktor, 2018; Gezgin, 2018). Nevertheless, Jeong, Kim, Yum, and Hwang (2016) researched if games or social networks predicted greater problematic smartphone use in participants and found that, although games and social networks were both predictors, the stronger predictor was social networks. Salehan and Negahban (2013) also found that social networks predicted problematic smartphone use. Further, these authors demonstrated that social network intensity and network size are important factors for predicting problematic smartphone use. Additionally, Sha, Sariyska, Riedl, Lachmann, and Montag (2019) demonstrated that there is a specific relation between problematic smartphone use and Whatsapp and Facebook use disorders. These authors argue that the problematic use of smartphones is more strongly related with Whatsapp use disorder than with Facebook use disorder and that this association is more likely to be present in women. Lastly, Sha et al. (2019) affirm that this relation is mediated through the fear of missing out.

Other studies have tried to predict problematic smartphone use (e.g., Bian & Leung, 2014; Kim et al., 2016; Lachmann, Duke, Sariyska, & Montag, 2019; Peterka-Bonetta et al., 2019). Kim et al. (2016) used logistical regression to demonstrate that demographic variables (gender, age, education level, occupation, marital status), smartphone use (weekday average usage hours, weekend average usage hours), and personality factors (behavioral inhibition system, behavioral activation system, impulsivity, self-control) could predict problematic smartphone use predisposition. Bian and Leung (2014) used multiple linear regression to predict problematic smartphone use by age, gender, grade, family monthly income, loneliness, shyness, and smartphone usage (e.g., information seeking, utility, fun seeking, sociability); they found that these variables predicted 20% of problematic smartphone use. Loneliness and shyness were the most predictive variables, while smartphone usage was the least. Peterka-Bonetta et al. (2019) used a hierarchical regression to predict problematic smartphone use with the following predictors: age, gender, the big five personality traits, anxiety, and impulsivity. They found that demographics predicted 5.23% of the smartphone use, the big five predicted 7.17%, and anxiety and impulsivity predicted 4.18%, totaling a prediction of 16.58% of the variance of the smartphone use. Furthermore, Lachmann et al. (2019) used a hierarchical regression to predict “problematic digital use,” a composite score between problematic Internet use and problematic smartphone use. Their final model had age, gender, self-directedness, and the big five personality traits as the predictors. Specifically, the demographic variables accounted for 2.6% of the variance in the problematic digital use, self-directness accounted for 15.6%, and the big five accounted for 5.0%, totaling for 23.2% of the variance explained.

Similarly, Mitchell and Hussain (2018) used multiple linear regression and found that age and personality/psychological traits (impulsiveness, extraversion, excessive reassurance, and depression) could predict problematic smartphone use; they found that the variance of these variables explained 23% of problematic smartphone use variance. Furthermore, Lee and Lee (2017) demonstrated that demographic variables (i.e., gender), attachment variables (i.e., attachment to parents), and school life motivations (i.e., obtaining infotainment, gaining peer acceptance, finding new people) are predictors of proneness to problematic smartphone use in middle and high school students; these variables predicted 27.1% of the variation of proneness to problematic smartphone use. Moreover, Durak (2018) found that demographic variables (i.e., gender, age, educational level), variables related to parents (i.e., mother’s education level), information technology usage variables (i.e., Internet usage experience, daily Internet usage time), smartphone usage variables (i.e., smartphone control frequency, daily smartphone usage time, smartphone usage experience, smartphone usage purpose), and school achievement variables (i.e., mathematics achievement, science achievement, language lesson achievement, social sciences achievement, information technology achievement) could predict problematic smartphone use; these variables explained almost 50% of the problematic smartphone use variance in secondary and high school students in Turkey.

Notably, there is common ground in these regression studies. Participant age and sex were used as variables in almost every study. Additionally, economic status was measured by family monthly income, mother’s education level, education level, and occupation. Some studies focused on psychological aspects such as loneliness, shyness, personality traits, and attachment styles (e.g., Lachmann et al., 2019; Peterka-Bonetta et al., 2019). These variables are all in accordance with the I-PACE model (Brand et al., 2016; 2019), specifically the person variables. Additionally, studies (e.g., Durak, 2018) have considered the content participants access on their smartphones (i.e., smartphone usage, smartphone usage experience, smartphone usage purpose) and time spent on smartphones; however, time spent is not a good measure because it does not provide information about what the participant is doing during that time (Panova & Carbonell, 2018).

Although previous studies identified some problematic smartphone use predictors, most of them studied elementary and high school students (e.g., Lee & Lee, 2017; Durak, 2018). Additionally, only a few models tried to relate social apps (social network apps and messaging apps), despite that they are an important aspect of problematic smartphone use. Finally, to the best of our knowledge, no study created models using the smartphone model as a predictor, even though this may be an important variable for problematic smartphone use (Hope, 2010). Therefore, the objective of this study was to identify predictors of problematic smartphone use in university students among demographic characteristics, loneliness, social app use, and smartphone model.

To reach this objective, we selected variables that have already been studied in the literature and added other variables that have not been studied but may be related to smartphone use. The variables are age, gender, family income, university major, smartphone model/operating system, social networks used, the importance attributed to smartphone social apps, and loneliness. The relevance of this type of study is due to a greater need to understand the relationship between problematic smartphones use and problematic social network use. As most recent theories point out (Montag et al., 2019), the two concepts probably overlap within the problematic Internet use, in which case it is necessary to raise more evidence, especially in cultures that escape the rich and industrialized context, as is the case in most research reported. Furthermore, it is important to understand how the use of smartphones can be healthy and productive.

Based on previous research, we hypothesized the following:

1. Age was negatively correlated with smartphone use (Bian & Leung, 2014; Kim et al., 2016).
2. Women would have a higher score on problematic smartphone use (Lee et al., 2017).
3. No relation would be found between family incomes and smartphone use (Aktürk et al., 2018; Alhassan et al., 2018; Aljomaa et al., 2016; Cha & Seo, 2018).
4. No relation would be found between university majors and smartphone use (Sanal & Ozer, 2017).
5. Users of iPhone/iOS would demonstrate more problematic smartphone use (Hope, 2010).
6. More use of different social network apps was related with higher smartphone usage (Arnavut et al., 2018; Gezgin, 2018; Haug et al., 2015; Kircaburun & Griffiths, 2018).
7. The importance attributed to the social networks was directly correlated with problematic smartphone use (Arnavut et al., 2018; Gezgin, 2018; Haug et al., 2015).
8. And, loneliness will be positively correlated with problematic smartphone use (Bian & Leung, 2014; Darci et al., 2015).

This is one of the first studies on smartphone use originating in Brazil as well as South America. Given that this is a problem that has gained popularity due to its importance, data from this region of the world are missing, which makes this study highly relevant.

## Methods

This cross-sectional study was conducted between August and September 2017 at Mackenzie Presbyterian University, a private university located in the city of São Paulo, Brazil. The study was approved by the Mackenzie Presbyterian University ethics committee. All study participants provided written informed consent.

### Participants

The sample consisted of 257 university students from Mackenzie Presbyterian University. The students were invited to participate in the study through posts on the University’s social network groups or through invitations of colleagues. We included Brazilian students who were enrolled in any undergraduate or post-graduate course and who were at least 18 years old. Students who did not own or use a smartphone or who were unable to read/understand written questionnaires in Portuguese were excluded, as well as those with a history of any psychiatric or neurological disorder. We chose to exclude participants with a history of psychiatric or neurological disorders because it could influence the measure of loneliness or the understanding of questions in the study. In exchange for participation, the students received a specific type of course credit, which is necessary to graduate from this university.

### Questionnaires

The participants answered three written questionnaires: the Smartphone Addiction Scale (SAS) (Kwon et al., 2013), a questionnaire on smartphone usage patterns (QSUP) created by the researchers specifically for this study, and the Brazilian version of the UCLA-R Loneliness Scale (Barroso, Andrade, Midgett, & Carvalho, 2016). All participants answered these questionnaires in the neuroscience laboratory of Mackenzie Presbyterian University, in São Paulo, Brazil.

We opted to use the SAS because we wanted an instrument that captured many facets of problematic smartphone use. Different from the smartphone addiction inventory, the SAS also captures behaviors related to cyberspace-oriented relationships (Lin et al., 2014). The questions used in the QSUP were based on behaviors that could possibly relate to problematic smartphone use. We created this questionnaire because there was no published questionnaire related to smartphone habits. Lastly, the UCLA-R Loneliness Scale was selected to measure loneliness because other researchers (e.g., Bian & Leung, 2014; Aktürk et al., 2018) have used it, providing us with comparative results. These instruments are discussed below.

#### Brazilian version of the smartphone addiction scale (SAS-BR)

We used the Brazilian Portuguese version (Busin, 2018) of the SAS (SAS-BR), which was originally created by Kwon, Kim, et al. (2013). The SAS measures the smartphone usage of participants. It has 33 items with six Likert-type answers (1 = “strongly disagree” and 6 = “strongly agree”). Possible scores range from 33 to 198; higher scores indicate higher degrees of problematic smartphone use. Notably, the SAS was created before concerns arose about whether smartphone addiction is really an addiction (Panova & Carbonell, 2018). Nevertheless, because it primarily evaluates smartphone usage aspects, it can be used as a tool to investigate problematic smartphone usage (Kwon, Lee, et al., 2013). In this study, the internal consistency (Cronbach’s alpha) of the SAS-BR was excellent (*α* = 0.93).

#### Questionnaire on smartphone usage patterns (QSUP)

This self-responsive, multiple-choice questionnaire was developed by the authors to assess characteristics of the participants and their smartphones, and their usage of social apps (social network apps and messaging apps) through the device. The first part of this questionnaire collected data on participants’ age, gender, family monthly income, university major, smartphone model/system, and the approximate value of their device. The second part of the questionnaire inquired about specific social apps (WhatsApp, Facebook, Instagram, Snapchat) they accessed through their smartphone and if the participants used their smartphone for work purposes. They then answered a question asking what degree of importance (ranging from 1 = “not important” to 5 = “very important”) they attributed to each social app and to the use of the device for work purposes.

#### Brazilian version of the UCLA-R loneliness scale (UCLA-BR)

We assessed participants’ loneliness using the Portuguese Brazilian version of the UCLA Loneliness Scale (Barroso, Andrade, & Oliveira, 2016) (UCLA-BR). The UCLA-R scale consists of 20 items with four Likert-type answers (0 = “never” to 3 = “frequently”). Total scores range from 0 to 60; higher scores indicate higher levels of loneliness. We used the total UCLA-BR score cut-offs proposed by Barroso, Andrade, and Oliveira (2016): < 23 (minimal loneliness), 23 to 35 (mild loneliness), 36 to 47 (moderate loneliness), and 48 to 60 (intense loneliness). In this study, the internal consistency (Cronbach’s alpha) of the UCLA-BR was of 0.94, indicating an excellent internal consistency.

### Data collection

This study was carried out in accordance with all the recommendations of the Ethics Committee of Research of the Mackenzie Presbyterian University. Participants gave written informed consent. The protocol was approved by the Ethics committee under CAAE number 98608718.0.0000.0084. The data collection happened in the Social and Cognitive Neuroscience Laboratory, in Mackenzie Presbyterian University of São Paulo, Brazil. Participants went to the laboratory and were placed in a room with a small group of other participants. The maximum number of participants per room was 5. Participants were placed within some distance from each other, such that they could not see the answer of other participants. They answered the QSUP, the SAS-BR, and the UCLA-BR. After finishing, participants called the researchers to inform them that they had finished. Participants received course credits, as required by the university so they can graduate, in exchange for their participation. The course credits were given upon the end of the data collection.

### Statistical analyses

First, we tested the normality distribution of the SAS-BR scores. To do this, we used the Shapiro-Wilk test and analyzed the skewness and kurtosis. We present participants’ characteristics using means and standard deviations (SD), percentages, and minimum and maximum values. We used Student’s *t* tests (for items with two possible answers; Welch’s *t* tests were conducted when Levene’s test was violated) and one-way analysis of variance (ANOVA) (for items with three or more possible answers) to assess differences in mean smartphone usage scores (SAS-BR) according to participant characteristics. Analysis of covariance (ANCOVA) was conducted to investigate if age and sex could be mediators of the analysis made with *t* tests and ANOVAS. We used Spearman’s rank correlation coefficient to assess the correlation between participants’ smartphone use scores (SAS-BR) and the importance they attributed to social apps. To assess the correlation between smartphone use scores (SAS-BR) and loneliness (UCLA-BR) scores, we used Pearson’s correlation coefficient. Lastly, we conducted a hierarchical multiple regression to investigate the influence of demographic characteristics, loneliness, smartphone social apps, and smartphone model on problematic smartphone use. R (R Core Team, 2018) was used for the hierarchical regression analysis, SPSS version 22 was used for all other analyses.

Because some of the predictors can be categorical variables, we transformed them into dummy variables (Field, Miles, & Field, 2012). The sex variable was categorized as “male” and “female,” with male coded as “0” and female as “1.” For categorical variables with more than two categories, Field et al. (2012) suggest using dummy variables with the group that represents most people as the reference. Family income was grouped in six categories, with “between 10,000 to 20,000 reais (BRL)” coded as the baseline; the model of the smartphone was grouped in three categories (iPhone, Samsung, others), with “iPhone” coded as the baseline. The number and percentage of participants in each category can be found in Table 1. The continuous variables were entered in the model as their original values.

**Table 1:** Participant characteristics, smartphone usage patterns, and smartphone addiction scores of 257 Brazilian university students

Characteristics	*n*	%	SAS-BR scores Mean ± SD		*p* value		Effect size
Gender				*t* = 3.886	< 0.001^a^	*d* = 0.54	Moderate
Female	187	72.8	101.74 ± 26.56				
Male	70	27.2	88.01 ± 24.69				
Family monthly income (BRL)^c^				*F* = 1.953	0.073^b^	*η*^2^ = 0.04	Small
< 2000	10	3.9	104.00 ± 16.02				
2000–2999	16	6.2	94.75 ± 20.01				
3000–3999	26	10.1	93.77 ± 24.11				
4000–4999	24	9.3	94.79 ± 23.82				
5000–9999	59	23.0	90.34 ± 27.88				
10,000–20,000	69	26.8	104.33 ± 26.07				
> 20,000	53	20.6	101.68 ± 30.22				
University major				*F* = 0.400	0.753^2^	*η*^2^ < 0.01	NS
Humanities	124	48.2	98.04 ± 27.08				
Natural sciences	43	16.7	94.49 ± 21.96				
Formal and applied sciences	7	2.7	103.14 ± 26.00				
Not informed	83	32.3	99.34 ± 28.69				
Smartphone model				*F* = 10.850	< 0.001^2^	*η*^2^ = 0.08	Moderate
iPhone	147	57.2	104.33 ± 27.20				
Samsung	61	23.7	91.75 ± 22.47				
Others	49	19.1	86.82 ± 25.08				
Uses WhatsApp				-	-	-	-
Yes	257	100	98.00 ± 26.73				
No	0	0	-				
Uses Facebook				*t* = 1.259	0.295^1^	*d* = 0.35	Small
Yes	246	95.7	98.37 ± 26.90				
No	11	4.3	89.73 ± 22.06				
Uses Instagram				*t* = 5.716	< 0.001^1^	*d* = 0.92	Large
Yes	219	85.2	101.21 ± 26.40				
No	38	14.8	79.53 ± 20.64				
Uses Snapchat				*t* = 3.623	< 0.001^1^	*d* = 0.46	Small
Yes	109	42.4	104.86 ± 25.84				
No	148	57.6	92.95 ± 26.23				
Uses smartphone for work^d^				*t* = 0.592	0.554^1^	*d* = 0.08	NS
Yes	189	73.5	98.63 ± 26.70				
No	67	26.1	96.37 ± 27.11				

*BRL* Brazilian Reais, *SAS-BR* Brazilian Portuguese version of the Smartphone Addiction Scale, *SD* Standard deviation

^a^Student’s *t* test

^b^One-way ANOVA

^c^At the time of the study, 1 BRL ≅ 0.315 USD

^d^One participant did not answer

The multiple regression model steps were created as follows: participants’ age, sex, and family income were entered in the first step (demographic characteristics); loneliness scores were entered in the second step (loneliness); Facebook importance, WhatsApp importance, and Instagram importance were entered in the third step (smartphone social app importance); and the model of the smartphone was entered in the fourth and final step (smartphone model). All standardized coefficients were reported in each step for each variable, and the collinearity was reported for the last model.

## Results

### Participant characteristics and smartphone usage patterns

The SAS-BR score was normally distributed (Shapiro-Wilk test = .991, *p* = .107, Skewness = 0.272, Skewness SE = 0.152, Kurtosis = − 0.042, Kurtosis SE = 0.303). Table 1 presents the main characteristics of the 257 participants. Most were women, had an average family monthly income of at least 5000 BRL (1577 USD), and were humanities majors. Their mean age was 22.4 (standard deviation, SD, 3.8) years, but participants’ age ranged from 18 to 38 years. Most participants owned an iPhone, which they had purchased themselves. Almost three quarter of the participants used their smartphone for work purposes. The participants estimated that the average (SD) cost of their current smartphone was 2024.50 (1083.90) BRL, which is approximately equivalent to 638.49 USD. Five percent (*n* = 13) either did not answer this question or did not know the price of their device.

The participants’ mean (SD) smartphone use score (SAS-BR) was 98.00 (26.73), out of a maximum of 198 points, with a range from 40 to 183. Their mean (SD) loneliness score (UCLA-BR) was 19.49 (12.50), out of a maximum of 60 points, with a range from 0 and 59. Nearly 63% (*n* = 162) of the participants had minimal loneliness, 24.5% (*n* = 63) had mild loneliness, 9.7% (*n* = 25) had moderate loneliness, and 2.7% (*n* = 7) had intense loneliness scores.

All participants stated that they used their smartphone to access WhatsApp, nearly 96% to access Facebook, 85% to access Instagram, and 42% to access Snapchat (Table 1). The participants attributed the highest mean score of importance (out of a maximum of 5.0) to WhatsApp (4.40, SD = 0.80, median = 5), followed by Facebook (3.18, SD = 0.98, median = 3), and Instagram (3.10, SD = 1.15, median = 3).

To ensure that effects found in Table 1 were not mediated through sex or age of the participants, ANCOVAs were conducted with these variables as covariates. The results suggested that family monthly income (*F* = 2.014, *p* = 0.065, ηp^2^ = 0.048), university major (*F* = 1.600, *p* = 0.190, ηp^2^ = 0.019), and smartphone usage for work (*F* = 0.448, *p* = 0.504, ηp^2^ = 0.002) did not predict problematic usage; however, smartphone model (*F* = 6.112, *p* = 0.003, ηp^2^ = 0.048), Facebook usage (*F* = 0.480, *p* = 0.489, ηp^2^ = 0.002), Instagram usage (*F* = 11.891, *p* = 0.001, ηp^2^ = 0.046), and Snapchat usage (*F* = 6.863, *p* = 0.009, ηp^2^ = 0.027) did. No results that were statistically significant in Table 1 became non-significant or vice versa, indicating that sex and age were not mediators.

### Smartphone usage, loneliness, and importance attributed to smartphone social apps correlations

Several correlations were conducted between SAS-BR, UCLA-BR, and importance attributed to smartphone-related activities. These correlations are shown in Table 2. SAS-BR was significantly positively correlated with UCLA-BR (*p* < 0.001) as well as importance attributed to WhatsApp (*p* < 0.001), Facebook (*p* < 0.001), Instagram (*p* < 0.001), and Snapchat (*p* = 0.012). We found no correlation between SAS-BR scores and the importance attributed to use of the smartphone for work purposes (*p* = 0.155).

**Table 2:** Correlation table of the correlations between SAS-BR scores, UCLA-BR scores, social apps importance scores, and using smartphone for work importance

	1.	2.	3.	4.	5.	6.	7.
1. SAS-BR	1.00	0.30***^a^	0.41***^b^	0.38***^b^	0.30***^b^	0.24*^b^	0.10^b^
2. UCLA-BR		1.00	− 0.08^b^	0.14*^b^	0.01^b^	0.01^b^	− 0.09^b^
3. WhatsApp Importance			1.00	0.42***^b^	0.23***^b^	0.04^b^	0.23**^b^
4. Facebook Importance				1.00	0.33***^b^	0.30***^b^	0.03^b^
5. Instagram Importance					1.00	0.41***^b^	− 0.08^b^
6. Snapchat Importance						1.00	− 0.11^b^
7. Work Importance							1.00

*SAS-BR* Brazilian Version of Smartphone Addiction Scale, *UCLA-BR* Brazilian Version of UCLA Loneliness Scale

*p < .05; **p < .01; ***p < .001

^a^Pearson correlation coefficient

^b^Spearman´s rank correlation coefficient

### Predicting smartphone usage based on demographic characteristics, smartphone social apps, loneliness, and smartphone model

To examine the association of these variables with the SAS-BR score, a hierarchical multiple linear regression analysis was conducted. The regression predictors, standardized and unstandardized coefficients, standard error, tolerance, and prediction percentage are shown in Table 3. All models demonstrated a significance level less than .001. The demographic characteristics step predicted 9.9% of the SAS-BR variance. The loneliness step increased the prediction by an additional 7.2%. When adding social network importance, the independent variables predicted an additional 13.1% of the variance. Finally, the last model (multiple *R* = 0.57)—with demographic characteristics, loneliness, social apps importance, and smartphone model—predicted 32.2% of the SAS-BR variance.

**Table 3:** Hierarchical regression analysis predicting problematic smartphone use, their standardized and unstandardized coefficients, the standard error (SE), and tolerance

Predictor	Step 1	Step 2	Step 3	Step 4	*b*^a^	SE^a^	Tolerance^a^
	*β*	*β*	*β*	*β*			
Intercept	-	-	-	-	43.03	15.99	
Demographic characteristics							
Age	− 0.19**	− 0.15*	− 0.11	− 0.11	− 0.84	0.47	0.88
Sex (female)	0.19**	0.19**	0.08	0.06	3.94	3.72	0.94
Family monthly income (BRL)							
< 2000	− 0.02	− 0.05	− 0.02	0.01	0.32	8.07	0.85
2000–2999	− 0.05	− 0.07	− 0.06	− 0.04	− 4.73	7.63	0.86
3000–3999	− 0.13*	− 0.14*	− 0.02	− 0.02	− 1.54	5.76	0.76
4000–4999	− 0.11	− 0.11	− 0.02	0.02	1.49	6.06	0.79
5000–9999	− 0.22**	− 0.20**	− 0.11	− 0.11	− 6.76	4.41	0.70
> 20,000	− 0.04	− 0.01	− 0.01	− 0.02	− 1.50	4.37	0.67
Loneliness							
UCLA-BR		0.28***	0.31***	0.31***	0.65	4.41	0.91
Smartphone social app importance							
WhatsApp importance			0.28***	0.26***	9.23	2.36	0.77
Facebook importance			0.10	0.11	2.86	1.80	0.72
Instagram importance			0.20**	0.19**	4.40	1.42	0.83
Smartphone type							
Smartphone model							
Samsung				− 0.01	− 0.87	4.17	0.79
Others				− 0.17**	− 12.31	4.54	0.80
Adjusted *R*^2^	.099***	.171***	.302***	.322***			
Δ*R*^2^		.072	.131	.020			

*BRL* Brazilian Reais, *SAS-BR* Brazilian Version of Smartphone Addiction Scale, *UCLA-BR* Brazilian Version of UCLA Loneliness Scale

*p < .05; **p < .01; ***p < .001

^a^Measure of the last model

## Discussion

We found a positive correlation between problematic smartphone use in Brazilian university students and the importance they attributed to social media as well as between SAS-BR scores and loneliness scores. The magnitude of this effect was moderate. Although some studies (Darcin et al., 2016; Aktürk et al., 2018) did not find a correlation between problematic smartphone use and loneliness among university students, our findings suggest that there is a relationship between these two variables, in the same direction found in some prior studies (Bian & Leung, 2014; Darcin et al., 2015). Bian and Leung (2014) suggest that smartphones are a way for lonely people to alleviate loneliness, therefore leading lonely participants to use their smartphone more. Our results support that finding.

Additionally, we found that persons with certain characteristics (being female, owning an iPhone, and being an Instagram or Snapchat user) had higher SAS-BR scores. On the other hand, we did not detect differences in smartphone use scores according to the students’ university major, which is consistent with research by Sanal and Ozer (2017).

Although we did not detect statistically significant differences in smartphone use scores according to family income, this variable showed a bimodal trend (*p* < 0.10): users in the lowest (< 2000 BRL) and highest (> 10,000 BRL) family income strata had higher SAS-BR scores, indicating a greater likelihood of problematic smartphone use. Similarly, several other studies (Alhassan et al., 2018; Aljomaa et al., 2016; Cha & Seo, 2018) found no significant differences in problematic smartphone use according to users’ income, and Aktürk et al. (2018) reported similar results in a sample of high school students. Two possible hypotheses may help to explain our findings. First, participants with the highest family incomes have more money to spend on better smartphones in terms of functionality, and they may also have more free time because they may not need to work to contribute to their family’s income. The combination of these two factors (having a better smartphone and more free time) may have contributed to higher SAS-BR scores for the participants with higher family incomes. By contrast, the higher SAS-BR scores seen in our participants with the lowest family incomes may reflect a more limited social life (due to economic constraints) and the fact that they may compensate this by using their smartphones more intensely to maintain social relations (Aktürk et al., 2018).

Our finding that women have higher smartphone scores, indicating a greater likelihood of problematic smartphone use, than men is consistent with the literature (Lapointe et al., 2013). Lee et al. (2017) reported that being a woman was a risk factor for smartphone addiction, and Kwon, Kim, et al. (2013) even proposed that SAS score cut-off point should be higher for women than for men. Billieux, Van der Linden, d’Acremont, Ceschi, and Zermatten (2007) and Kwon, Lee, et al. (2013) suggest that women’s higher scores may be because women are more willing to interact socially than men. Additionally, Kwon, Kim, et al. (2013) highlight that women tend to be more aware and express their problems more openly than men in self-reporting instruments, which may explain the difference between their mean scores.

The smartphone use scores of our participants who owned an iPhone were significantly higher than of those who owned a Samsung or other smartphone model. Hope (2010) reported that a significant proportion of American university students who owned iPhones showed signs of problematic use, considered their smartphones more important than other essential personal items, and would perceive the loss of their mobile as a tragedy. Additionally, iPhone users are more likely to see their smartphone as a social status symbol (Shaw et al., 2016). In Brazil, iPhones usually are much more expensive than those with the Android system, such as Samsung phones. Therefore, it is possible that students who have problematic smartphone use may view their devices as a status symbol and be willing to pay more for it. However, more research is needed to investigate why this relationship exists.

In addition, participants who used Instagram or Snapchat had significantly higher SAS-BR scores. A previous study found that addiction to Instagram is related to increased daily Internet use (Kircaburun & Griffiths, 2018). Because people with problematic smartphone use utilize their phones a lot, it is possible that they use more types of social apps, such as Instagram and Snapchat, to entertain themselves and prolong the time that they spend on their device. This may be why we did not find significantly higher smartphone use scores in participants who used the most popular social apps (e.g., WhatsApp and Facebook). Of course, these relationships (between high SAS-BR scores and model and apps) may not be causal. For instance, it is possible that because iPhones are high-quality devices, a person who owns this model may have a higher risk of problematic smartphone use. However, it is also possible that a person with problematic smartphone use prefers to own an iPhone (i.e., a better device) to satisfy his/her needs. Similarly, it is possible that the use of social networks may increase the likelihood of problematic smartphone use or that people with problematic smartphone use may seek out more social networks to fulfill their needs.

We found significant correlations between smartphone use scores and the importance attributed to social apps by the participants. The correlation between smartphone use scores and the importance attributed to Snapchat was negligible, and those for WhatsApp, Facebook, and Instagram were low. The importance attributed to using the device for work purposes was not statistically correlated to smartphone use scores. Others have previously reported a connection between smartphones use and use of social networks (Haug et al., 2015; Arnavut et al., 2018; Gezgin, 2018; Sha et al., 2019). However, we did not find any previous studies on the importance attributed to specific social apps. The low correlations found in our study can be explained by the fact that problematic smartphone use is not linked just to social apps, but also to gaming, Internet browsing, and other activities (Jeong et al., 2016; Panova & Carbonell, 2018). Thus, social apps represent only a part of the smartphone use pattern and demonstrate a low correlation with the smartphone use score. Sha et al. (2019) found that problematic smartphone use was strongly linked with problems in Whatsapp use rather than problems in Facebook use. Indeed, our results points to the same conclusion. The correlation between the smartphone use and the importance given to Whatsapp was higher than the correlation with the importance given to other social networks.

Problematic smartphone use was more evident in participants who used less popular social apps (Instagram and Snapchat) and not necessarily related to the importance they attributed to these apps. Therefore, it seems that the use of additional social apps is perhaps more relevant to problematic smartphone use than the importance the users attribute to these social apps. In addition, the importance of the apps may change over time. For example, we hypothesize that users attribute more importance to the social apps in which they have the most friends or followers. As a result, the importance attributed to these social apps will change over time based on changes in numbers of users of these apps. These factors should be considered in future research examining social apps. In our study, the most important social network was Facebook, which was also the network with the most users in the study period (Statista, 2019). However, social networks like Instagram are gaining more users (Statista, 2018), which may generate a new trend in the coming years.

As expected, we found no correlation between SAS-BR scores and the importance attributed to the use of smartphones for work purposes. This is an expected result because problematic smartphone use is generally associated with pleasurable activities, such as using the Internet, social apps, or playing games (Panova & Carbonell, 2018), instead of work activities, which may not be as enjoyable.

In terms of the hierarchical regression, demographics characteristics (i.e., age, sex, and family monthly income) were predictors of problematic smartphone use. Specifically, being young, being female, and having a family monthly income higher than 10,000 BRL were related to more problematic smartphone use. These results are consistent with findings in previous studies (Kim et al., 2016; Lee & Lee, 2017; Durak, 2018; Mitchell & Hussain, 2018). Loneliness also predicted problematic smartphone use. The importance attributed to smartphone social apps also predicted problematic smartphone use: the higher the importance attributed to the social apps, the higher the likelihood of problematic smartphone use. Adding the importance attributed to social apps to the model provided the highest increase in the model’s predictive ability of problematic smartphone use. However, adding the smartphone model increased the model’s predictive ability only slightly, indicating that the use of an iPhone is not a strong predictor of problematic smartphone use. These results are intriguing because they show that when we evaluate the smartphone model separately, iPhone users present a higher SAS-BR mean, but when the smartphone model is inserted into a model that controls for age, sex, monthly family income, loneliness, and importance of social apps, this variable does not have great predictability for problematic smartphone use, although it is still significant. Specifically, adding the smartphone model into the model only increases the predictability by 2.0%, indicating that part of this variable’s importance may be diluted by the other variables in the model. This should be considered when designing future studies.

Our regression model predicted 32.2% of problematic smartphone use, which indicates that other variables may play a role in predicting problematic smartphone use. Future studies should examine other potential factors. For example, Lee and Lee (2017) demonstrated that attachment variables were predictors of problematic smartphone use, and Mitchell and Hussain (2018) and Peterka-Bonetta et al. (2019) independently showed that personality/psychological traits could also predict problematic smartphone use. Therefore, we strongly suggest that further studies consider other variables related to psychological styles and traits.

With respect to the I-PACE model (Brand et al., 2017; Brand et al., 2019), the present study works in two of its aspects: the person variables and the affective variables. Variables such as age, sex, family income, and loneliness fall under the first category of variables, whereas smartphone social app importance would mainly fall into the second category. In this sense, this study brings new evidence to light for this model.

Our results can be interpreted as follows: different characteristics can influence the use of smartphones. With the use of statistical analysis, we were able to identify that some of the most important ones are loneliness, the use of different social networks, and the importance given to these social networks. This is expected, given that part of the smartphone use is correlated with the need to connect with other people and that one of the main activities on the smartphone is to use social networks. However, our results still leave room for other measures that may interfere with smartphone use. One of them may be the fear of missing out, a measure that has been extensively investigated recently. Montag, Wegmann, et al. (2019) describe a mechanism that explains how the fear of missing out can be used as a moderator of smartphone use. This measure should be used in new models.

This study had several limitations, which can guide future research. First, we included only university students; therefore, more studies are needed to confirm our findings in other populations. Second, it is important to acknowledge that our sample was small, generating smaller power in our results. With this in mind, further studies should have more participants to generate greater statistical power. Third, we should emphasize that our results were of correlational nature and, therefore, no causal inference can be made based on our results. Fourth, our study did not measure social network addiction/maladaptive use, which could be an interesting measure because problematic use may be caused by social network usage. Therefore, future similar studies should incorporate scales that measure social network addiction/maladaptive use. More research is also needed to further explore how family income and economic status are related to smartphone addiction. These studies should specify this as one of their objectives and calculate an adequate sample size to answer this question.

Our results have theoretical and practical implications. First, our study is one of the first to study problematic smartphone use in Brazil and in South America. Second, we found a weak correlation between problematic smartphone use and the importance attributed to smartphone social apps. In addition, we found that certain factors (being a woman, owning an iPhone, and being an Instagram or Snapchat user) increase the likelihood of problematic smartphone use. Our results can be useful for clinical psychiatrists in the management of patients struggling with smartphone use and to identify high-risk groups. Finally, our results shed new light on some of the predictors of problematic smartphone use; we found that demographics characteristics such as age, sex, and family monthly income, loneliness, and social app importance were predictors of problematic smartphone use, but the smartphone model was less important in this model.

## Conclusion

In sum, this study produced interesting findings for problematic smartphone use. First, one of the biggest predictors of problematic smartphone use was the importance attributed to social network apps, even when loneliness was controlled. This indicates that the relationship between problematic smartphone use and social network use was the most important relationship we examined. Additionally, the results suggest that the smartphone model (iPhone/Samsung/other) is not particularly relevant in predicting problematic smartphone use. Furthermore, this study was carried in Brazil and is one of the first studies from South America on smartphone usage. Lastly, this manuscript contributes to advancing the models contained in other scientific articles. Most prior models included different variables, with some common ground. In this study, the common variables used in other studies were examined as well as additional measures. Future studies should consider including socioeconomic measures, loneliness measures, and social network usage/importance measures as well as adding new variables, such as personality traits.

## Data Availability

The datasets used and analyzed during the current study are available from the corresponding author on request.

## Abbreviations

ANCOVA: Analysis of covariance
ANOVA: Analysis of variance
BRL: Reais
I-PACE model: Interaction of Person-Affect-Cognition-Execution model
QSUP: Questionnaire on smartphone usage patterns
SAS: Smartphones Addiction Scale
SAS-BR: Brazilian version of Smartphone Addiction Scale
SD: Standard deviation
SE: Standard error
UCLA-BR: Brazilian version of the UCLA-R Loneliness Scale

## References

[CR1] Aktürk Ü, Budak F, Gültekin A, Özdemir A (2018). Comparison of smartphone addiction and loneliness in high school and university students. Perspectives in Psychiatric Care.

[CR2] Alhassan AA, Alqadhib EM, Taha NW, Alahmari RA, Salam M, Almutairi AF (2018). The relationship between addiction to smartphone usage and depression among adults: A cross sectional study. BMC Psychiatry.

[CR3] Aljomaa SS, Al Qudah MF, Albursan IS, Bakhiet SF, Abduljabbar AS (2016). Smartphone addiction among university students in the light of some variables. Computers in Human Behavior.

[CR4] Alosaimi FD, Alyahya H, Alshahwan H, Al Mahyijari N, Shaik SA (2016). Smartphone addiction among university students in Riyadh, Saudi Arabia. Saudi Medical Journal.

[CR5] Andrew, O. (2018). The history and evolution of the smartphone: 1992-2018. Text request. Available at: https://www.textrequest.com/blog/history-evolution-smartphone/ (Acessed 29 May 2019).

[CR6] Arnavut A, Nutri C, Direktor C (2018). Examination of the relationship between phone usage and smartphone addiction based on certain variables. Annals of Psychology.

[CR7] Barroso SM, Andrade VS, Midgett AH, Carvalho RGN (2016). Evidências de validade da Escala Brasileira de Solidão UCLA [evidence of validity of the Brazilian scale of loneliness UCLA]. Brazilian Journal of Psychiatry.

[CR8] Barroso SM, Andrade VS, Oliveira NR (2016). Escala Brasileira de Solidão: Análises de Resposta ao item e definição dos pontos de corte [Brazilian loneliness scale: Item response analysis and definition of cut-off points]. Brazilian Journal of Psychiatry.

[CR9] Bian M, Leung L (2014). Linking loneliness, shyness, smartphone addiction symptoms, and patterns of smartphone use to social capital. Social Science Computer Review.

[CR10] Billieux J, Philippot P, Schmid C, Maurage P, De Mol J, Van der Linden M (2015). Is dysfunctional use of the mobile phone a behavioural addiction? Confronting symptom-based versus process-based approaches. Clinical Psychology & Psychotherapy.

[CR11] Billieux J, Van der Linden M, d’Acremont M, Ceschi G, Zermatten A (2007). Does impulsivity relate to perceived dependence on and actual use of the mobile phone?. Applied Cognitive Psychology.

[CR12] Boumosleh JM, Jaalouk D (2017). Depression, anxiety, and smartphone addiction in university students- A cross sectional study. PLoS One.

[CR13] Brand M, Wegmann E, Stark R, Müller A, Wölfling K, Robbins TW, Potenza MN (2019). The interaction of person-affect-cognition-execution (I-PACE) model for addictive behaviors: Update, generalization to addictive behaviors beyond internet-use disorders, and specification of the process character of addictive behaviors. Neuroscience & Biobehavioral Reviews.

[CR14] Brand, M., Young, K. S., Laier, C., Wölfling, K., & Potenza, M. N. (2016). Integrating psychological and neurobiological considerations regarding the development and maintenance of specific internet-use disorders: An interaction of person-affect-cognition-execution (I-PACE) model. *Neuroscience & Biobehavioral Reviews*, *71*, 252–266. 10.1016/j.neubiorev.2016.08.033. 10.1016/j.neubiorev.2016.08.033 27590829

[CR15] Busin Y (2018). Emotional state and pattern of use of social networks: Instrument development and analysis of the effect of negative emotions in the attribution of financial and sentimental values [Estado emocional e padrão de uso de redes sociais: Desenvolvimento de instrumento e análise do efeito de emoções negativas na atribuição de valores financeiros e sentimentais]. Developmental disorders PhD [thesis].

[CR16] Cha SS, Seo BK (2018). Smartphone use and smartphone addiction in middle school students in Korea: Prevalence, social networking service, and game use. Health psychology open.

[CR17] Chen J, Liang Y, Mai C, Zhong X, Qu C (2016). General deficit in inhibitory control of excessive smartphone users: Evidence from an event-related potential study. Frontiers in Psychology.

[CR18] Darcin AE, Kose S, Noyan CO, Nudermov S, Yilmaz O, Dilbaz N (2016). Smartphone addiction and its relationship with social anxiety and loneliness. Behavior and Information Technology.

[CR19] Darcin AE, Noyan C, Nurmedov S, Yilmaz O, Dilbaz N (2015). Smartphone addiction in relation with social anxiety and loneliness among university students in Turkey. European Psychiatry.

[CR20] de Cock R, Vangeel J, Klein A, Minotte P, Rosas O, Meerkerk G (2014). Compulsive use of social networking sites in Belgium: Prevalence, profile, and the role of attitude toward work and school. Cyberpsychology, Behavior and Social Networking.

[CR21] Durak, H. Y. (2018). *Investigation of nomophobia and smartphone addiction predictors among adolescents in*. Turkey: Demographic variables and academic performance. The Social Science Journal. 10.1016/j.soscij.2018.09.003.

[CR22] Eide TA, Aarestad SH, Andreassen CS, Bilder RM, Pallesen S (2018). Smartphone restriction and its effect on subjective withdrawal related scores. Frontiers in Psychology.

[CR23] Elhai JD, Yang H, Fang J, Bai X, Hall BJ (2020). Depression and anxiety symptoms are related to problematic smartphone use severity in Chinese young adults: Fear of missing out as a mediator. Addictive Behaviors.

[CR24] Elhai, J. D., Yang, H., & Montag, C. (2019). Cognitive-and emotion-related dysfunctional coping processes: Transdiagnostic mechanisms explaining depression and anxiety’s relations with problematic smartphone use. *Current Addiction Reports*, 1-8. 10.1007/s40429-019-00260-4.

[CR25] Elhai, J. D., Yang, H., Rozgonjuk, D., & Montag, C. (2019). Using machine learning to model problematic smartphone use severity: The significant role of fear of missing out. *Addictive Behaviors*, *106261*. 10.1016/j.addbeh.2019.106261. 10.1016/j.addbeh.2019.106261 31901886

[CR26] Field A, Miles J, Field Z (2012). *Discovering statistics using R*.

[CR27] Fransson A, Chóliz M, Håkansson A (2018). Addiction-like mobile phone behavior – Validation and association with problem gambling. Frontiers in Psychology.

[CR28] Gezgin DM (2018). Understanding patterns for smartphone addiction: Age, sleep duration, social network use and fear of missing out. Cypriot Journal of Educational Science.

[CR29] Götz FM, Stieger S, Reips U-D (2017). Users of the main smartphone operating systems (iOS, android) differ only little in personality. PLoS One.

[CR30] Hadar A, Hadas I, Lazarovits A, Alyagon U, Eliraz D, Zangen A (2017). Answering the missed call: Initial exploration of cognitive and electrophysiological changes associated with smartphone use and abuse. PLoS One.

[CR31] Haug S, Castro RP, Kwon M, Filler A, Kowatsch T, Schaub MP (2015). Smartphone use and smartphone addiction among young people in Switzerland. Journal of Behavioral Addictions.

[CR32] Hope, D. (2010). iPhone addictive, Survey reveals. Live Science. Available at: http://www.livescience.com/6175-iphone-addictive-survey-reveals.html (Accessed 29 May 2019).

[CR33] Huang Y-T, Su S-F (2018). Motives for Instagram use and topics of interest among young adults. Future Internet.

[CR34] Jeong SH, Kim H, Yum JY, Hwang Y (2016). What type of content are smartphone users addicted to?: SNS vs. games. Computers in Human Behavior.

[CR35] Kim Y, Jeong JE, Cho H, Jung DJ, Kwak M, Rho MJ (2016). Personality factors predicting smartphone addiction predisposition: Behavioral inhibition and activation systems, impulsivity, and self-control. PLoS One.

[CR36] King DL, Herd MC, Delfabbro PH (2018). Motivational components of tolerance in internet gaming disorder. Computers in Human Behavior.

[CR37] Kircaburun K, Griffiths MD (2018). Instagram addiction and the big five of personality: The mediating role of self-liking. Journal of Behavioral Addictions.

[CR38] Kuss DJ, Griffiths MD (2017). Social networking sites and addiction: Ten lessons learned. International Journal of Environmental Research and Public Health.

[CR39] Kwon, M., Kim, D., Cho, H., & Yang, S. (2013). The smartphone addiction scale: Development and validation of a short version for adolescents. *PLoS One*, *8*(12). 10.1371/journal.pone.0083558. 10.1371/journal.pone.0083558 PMC3877074 24391787

[CR40] Kwon, M., Lee, J., Won, W., Park, J., Min, J., Hahn, C., … Kim, D. (2013). Development and validation of a smartphone addiction scale (SAS). *PLoS One*, *8*(2). 10.1371/journal.pone.0056936. 10.1371/journal.pone.0056936 PMC3584150 23468893

[CR41] Lachmann B, Duke É, Sariyska R, Montag C (2019). Who’s addicted to the smartphone and/or the internet?. Psychology of Popular Media Culture.

[CR42] Lapointe, L., Boudreau-Pinsonneault, C., & Vaghefi, I. (2013). Is smartphone usage truly smart? A qualitative investigation of IT addictive behaviors. 2013 46th Hawaii international conference on system sciences. 10.1109/hicss.2013.367.

[CR43] Lee C, Lee SJ (2017). Prevalence and predictors of smartphone addiction proneness among Korean adolescents. Children and Youth Services Reviews.

[CR44] Lee H, Kim JW, Choi TY (2017). Risk factors for smartphone addiction in Korean adolescents: Smartphone use patterns. Journal of Korean Medical Science.

[CR45] Lin YH, Chang LR, Lee YH, Tseng HW, Kuo TB, Chen SH (2014). Development and validation of the smartphone addiction inventory (SPAI). PLoS One.

[CR46] Mitchell L, Hussain Z (2018). Predictors of problematic smartphone use: An examination of the integrative pathways model and the role of age, gender, impulsiveness, excessive reassurance seeking, extraversion, and depression. Behavioral Sciences.

[CR47] Montag C, Lachmann B, Herrlich M, Zweig K (2019). Addictive features of social media/messenger platforms and freemium games against the background of psychological and economic theories. International Journal of Environmental Research and Public Health.

[CR48] Montag, C., Wegmann, E., Sariyska, R., Demetrovics, Z., & Brand, M. (2019). How to overcome taxonomical problems in the study of internet use disorders and what to do with "smartphone addiction"? *Journal of Behavioral Addictions*, 1–7. 10.1556/2006.8.2019.59. 10.1556/2006.8.2019.59 PMC8969715 31668089

[CR49] Oberst U, Wegmann E, Stodt B, Brand M, Chamarro A (2017). Negative consequences from heavy social networking in adolescents: The mediating role of fear of missing out. Journal of Adolescence.

[CR50] Oviedo-Trespalacios O, Nandavar S, Newton JDA, Demant D, Phillips JG (2019). Problematic use of mobile phones in Australia…is it getting worse? Front. Psychiatry.

[CR51] Panova T, Carbonell X (2018). Is smartphone addiction really an addiction?. Journal of Behavioral Addictions.

[CR52] Peterka-Bonetta, J., Sindermann, C., Elhai, J. D., & Montag, C. (2019). Personality associations with smartphone and internet use disorder: A comparison study including links to impulsivity and social anxiety. *Frontiers in Public Health*, *7*. 10.3389/fpubh.2019.00127. 10.3389/fpubh.2019.00127 PMC6579830 31245341

[CR53] Primack BA, Shensa A, Sidani JE, Whaite EO, Lin LY, Rosen D (2017). Social media use and perceived social isolation among young adults in the U.S. American Journal of Preventive Medicine.

[CR54] R Core Team. (2018). R: A language and environment for statistical computing. R Foundation for Statistical Computing, Vienna, Austria. http://www.R-project.org/.

[CR55] Salehan M, Negahban A (2013). Social networking on smartphones: When mobile phones become addictive. Computers in Human Behavior.

[CR56] Sanal, Y., & Ozer, Ö. (2017). Smartphone addiction and the use of social media among university students. Mediterranean Journal of Humanities, VII(2), 367-377. Doi:10.13114/MJH.2017.370.

[CR57] Sha P, Sariyska R, Riedl R, Lachmann B, Montag C (2019). Linking internet communication and smartphone use disorder by taking a closer look at the Facebook and WhatsApp applications. Addictive Behaviors Reports.

[CR58] Shaw H, Ellis DA, Kendrick L, Ziegler F, Wiseman R (2016). Predicting smartphone operating system from personality and individual differences. Cyberpsychology, Behavior and Social Networking.

[CR59] Statista. (2018). Number of monthly active Instagram users from January 2013 to June 2018 (in millions). In Statista - the statistics portal. Retrieved May 29, 2019, from https://www.statista.com/statistics/253577/number-of-monthly-active-instagram-users/

[CR60] Statista. (2019). Most popular social networks worldwide as of April 2019, ranked by number of active users (in millions). In Statista - the statistics portal. Retrieved May 29, 2019, from https://www.statista.com/statistics/272014/global-social-networks-ranked-by-number-of-users/

[CR61] Veissière SPL, Stendel M (2018). Hypernatural monitoring: A social rehearsal account of smartphone addiction. Frontiers in Psychology.

[CR62] Wegmann, E., Oberst, U., Stodt, B., & Brand, M. (2017). Online-specific fear of missing out and internet-use expectancies contribute to symptoms of internet-communication disorder. *Addictive Behaviors Reports*, *5*, 33–42. 10.1016/j.abrep.2017.04.001. 10.1016/j.abrep.2017.04.001 PMC5800583 29450225

